# Bradycardia, Renal Failure, Atrioventricular Nodal Blockade, Shock and Hyperkalemia (BRASH) Syndrome: A Clinical Case Study

**DOI:** 10.7759/cureus.34803

**Published:** 2023-02-09

**Authors:** Jaswanth R Jasti, Tirumala Nischal Jasty, Mohan Chandra Vinay Bharadwaj Gudiwada, Sahas Reddy Jitta

**Affiliations:** 1 Internal Medicine, University of South Dakota Sanford School of Medicine, Sioux Falls, USA; 2 Internal Medicine, Tower Health Medical Group, Reading, USA; 3 Internal Medicine, Advocate Illinois Masonic Medical Center, Chicago, USA; 4 Internal Medicine, Osmania Medical College, Hyderabad, IND

**Keywords:** shock, av nodal blockade, renal failure, bradycardia, brash syndrome

## Abstract

BRASH syndrome, which stands for Bradycardia, Renal failure, Atrioventricular (AV) Nodal blockade, and shock, is a relatively new clinical condition. Bradycardia develops because of the synergistic effect of AV-nodal blockers and hyperkalemia in a renal failure resulting in a vicious cycle of progressive bradycardia, renal hypoperfusion, and hyperkalemia. We present a case of an 88-year-old man with chronic systolic heart failure, atrial fibrillation, stage 3 chronic kidney disease, and dementia who presented to our emergency department with poor oral intake and weakness. He was found to have symptomatic bradycardia in the 30s secondary to hyperkalemia and beta-blockers in the setting of acute renal failure from dehydration, raising concern for BRASH syndrome. Treatment of each component conservatively resulted in complete resolution without the need for aggressive measures such as dialysis or pacing. This case report also discusses the pathophysiology, management, and the need for recognizing this underdiagnosed and novel clinical condition.

## Introduction

Bradycardia, Renal failure, Hyperkalemia, Atrioventricular Nodal (AV) blockade, and Shock collectively make up the acronym BRASH syndrome [1-3]. It is a relatively new and underdiagnosed clinical condition frequently confused with simple hyperkalemia. Most experienced clinicians have successfully treated BRASH syndrome without knowing its specific pathophysiology or labeling it as such. Indeed, most BRASH syndrome patients will improve with supportive therapy. However, understanding and recognizing BRASH syndrome as a distinct entity is crucial because it improves patient outcomes, especially in severe cases that may result in multi-organ failure [1].

## Case presentation

An 88-year-old man with dementia presented to our emergency department (ED) with one week of worsening confusion, poor oral intake, and generalized weakness. The patient's past medical history was significant for chronic systolic congestive heart failure (CHF), coronary artery disease (CAD), chronic atrial fibrillation (A-fib), stage 3 chronic kidney disease (CKD), type 2 diabetes mellitus, hypertension, hyperlipidemia, and hypothyroidism. Notable home medications at admission included carvedilol 25 mg twice daily, digoxin 0.125 mg once daily, quinapril 40 mg once daily, spironolactone 25 mg once daily, furosemide 20 mg once daily, metformin 500 mg twice daily and glipizide 5 mg twice daily.

**Figure 1:**
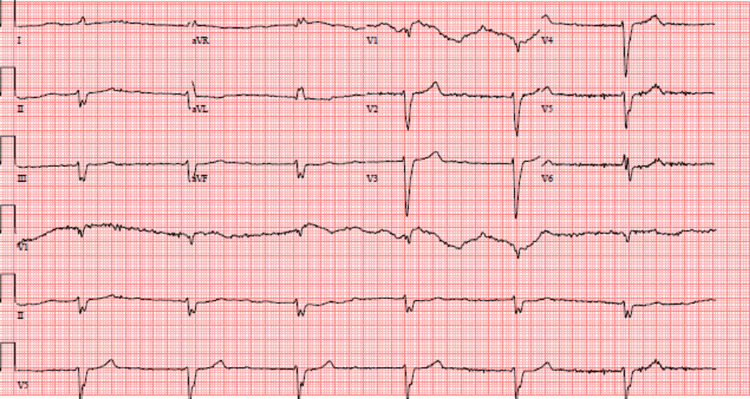
Admission EKG showing a heart rate of 38, junctional rhythm and a left bundle branch block. QTc (Bazett): 486 ms

The patient was only oriented to himself, with no agitation or acute distress. His heart rate was noted at 38 beats per minute, but his blood pressure was stable at 130/80 mm Hg. The rest of the vitals, including body temperature, respiratory rate, and peripheral oxygen saturation, were all within normal limits. A thorough review of the systems was not possible due to the patient's confusion, but the patient denied any chest pain, shortness of breath, palpitations, or headaches. His family denied that he had encountered any recent falls, syncope, or seizure-like events. Physical examination was unremarkable, with no focal deficits, clear lungs on auscultation, and regular S1 and S2 heart sound. Labs showed a glucose of 490 mg/dL, potassium 6.7 mEq/L, magnesium 2.7 mg/dL, creatinine 2.80 mg/dL, brain natriuretic peptide (BNP) 3080 pg/mL, initial troponin 0.168 ng/mL, and lactate 5.5 mmol/L (Table 1). A non-contrast CT of the head did not show any acute intracranial pathology. Chest X-ray did not show any evidence of pulmonary congestion or pleural effusion. EKG demonstrated bradycardia with a junctional rhythm and an old left bundle branch block (Figure 1), raising concern for BRASH syndrome.

**Table 1:** Laboratory values from admission and discharge with reference ranges Hgb: hemoglobin, WBC: white blood cells, BNP: brain natriuretic peptide, TSH: thyroid stimulating hormone

	Admission	Discharge	Reference
Hgb (g/dL)	9.8 (baseline 10-11)	11.2	13.5-17.5
WBC (10*3/uL)	6.8	6.7	4.0-11.0
Glucose (mg/dL)	490	199	70-100
Sodium (mEq/L)	135	138	137-145
Potassium (mEq/L)	6.7	4.1	3.5-5.1
Magnesium (mg/dL)	2.7	2.4	1.6-2.6
Chloride (mEq/L)	95	101	98-107
Bicarbonate (mEq/L)	17	27	22-30
Urea nitrogen (mg/dL)	75	22	6-22
Creatinine (mg/dL)	2.80 (baseline 1.5-1.8)	1.4	0.80-1.50
Lactate (mmol/L)	5.5	0.8	0.5-2.2
BNP (pg/mL)	3080 (baseline 300-650)	Not repeated	0-100
Troponin I (ng/ml)	peaked at 0.173	Not repeated	0.000-0.033
TSH (uIU/mL)	5.22	Not repeated	0.45-5.33
Free T4 (ng/dL)	0.9	Not repeated	0.7-1.5
Digoxin (ng/mL)	1.2	Not applicable	0.8-2.0

The patient was given a liter of normal saline bolus in the ED and was admitted to the intensive care unit on maintenance fluids with normal saline at 100 mL/hr. Home medications (i.e., carvedilol, furosemide, quinapril, spironolactone, metformin, glipizide, and tamsulosin) were all held. Hyperkalemia was managed with calcium gluconate, insulin + D50 fluid, and lokelma (sodium zirconium cyclosilicate) while being monitored on telemetry. Clinically, the patient continued to improve; his heart rate improved to the 70s, and he was stable enough to be transferred to the medical ward the following day. Lokelma was stopped once the hyperkalemia was corrected. While on intravenous fluids, the patient's renal function improved, and creatinine levels returned to normal. The patient was discharged to a skilled nursing facility (SNF) due to physical deconditioning and underlying dementia, with a resolution of admission laboratory findings (Table 1).

**Figure 2:**
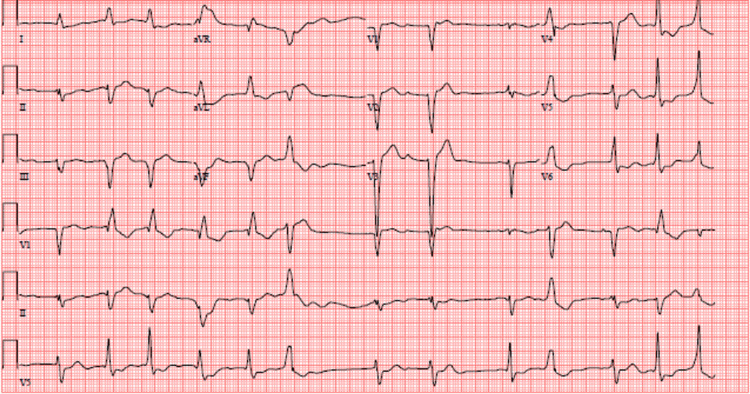
Follow-up EKG showing a heart rate of 79, atrial fibrillation and a left bundle branch block

At the time of discharge, the EKG revealed a baseline rhythm of atrial fibrillation, left bundle branch block, and non-peaked T-waves (Figure 2). Home carvedilol, hydralazine, and isosorbide nitrate that were started in the hospital were continued at discharge for chronic systolic heart failure, atrial fibrillation, and hypertension. Other home medications, such as quinapril, spironolactone, and digoxin, were discontinued, and the patient was advised to avoid non-steroidal anti-inflammatory drugs. Because of poorly controlled diabetes, metformin and glipizide were switched to a basal-bolus insulin regimen (Table 2). He was advised to see primary care and cardiology within a week.

**Table 2:** Notable medication list at admission and discharge TID: three times a day, BID: twice daily, OD: once daily, Prn: as needed, mg: milligram, mcg: microgram, U: units

Admission	Discharge
Carvedilol 25 mg BID	Carvedilol 6.25 mg BID
Quinapril 40 mg OD	Hydralazine 50 mg TID
Spironolactone 25 mg OD	Isosorbide dinitrate 40 mg OD
Digoxin 0.125 mg OD	Furosemide 20 mg OD
Furosemide 20 mg OD	Nitroglycerin prn
Nitroglycerin prn	Aspirin 81 mg OD
Aspirin 81 mg OD	Insulin glargine 10 U
Metformin 500 mg BID	Insulin aspart sliding scale
Glipizide 5 mg BID	Levothyroxine 125 mcg
Levothyroxine 125 mcg	

## Discussion

A BRASH syndrome is characterized by a clinical pentad of Bradycardia, Renal failure, AV-nodal blockade, shock, and hyperkalemia [4]. It is primarily a synergistic effect of hyperkalemia and medications that block the atrioventricular (AV) node in the setting of renal failure, resulting in a vicious cycle of progressive bradycardia and hypoperfusion worsening the renal failure and hyperkalemia to cause multi-organ failure and even death [1]. In order for pure hyperkalemia to cause bradycardia, pronounced elevations in potassium levels (>7) are typically required [5]. Patients with the BRASH syndrome may only have mild hyperkalemia, and the AV node blocker and hyperkalemia work together to cause bradycardia [6]. EKG findings are interesting because QRS widening or peaked T waves are usually not seen with BRASH.

Typically, the substrate consists of elderly patients with baseline renal dysfunction and underlying cardiac disease who are taking a beta-blocker or non-dihydropyridine calcium channel blockers, such as verapamil or diltiazem [1,7]. The most frequent cause of hypovolemia is gastroenteritis, inadequate oral intake, or dehydration during summer [1]. Other triggers include up-titrating beta-blockers or non-dihydropyridine calcium channel blockers or potassium-sparing diuretics (spironolactone), or any cause of kidney injury (e.g., dehydration, hepato-renal syndrome, or glomerulonephritis)[8]. 

The list of possible differentials in our patient presenting with generalized weakness and confusion and found to have an acute change in mental status, dry mucous membranes, and bradycardia in the setting of poor oral intake and underlying dementia is extensive, ranging from simple dehydration causing hyponatremia, acute kidney injury (AKI) on chronic kidney disease (CKD) to medication-induced, infections, hypothyroidism, stroke, congestive heart failure, and even acute coronary syndrome. Because the patient was afebrile, had a normal chest X-ray, and had a normal white blood cell count, infectious etiology was quickly ruled out. Urine analysis showed no evidence of infection. Decompensated hypothyroidism (myxedema) was also an unlikely cause because thyroid stimulating hormone (TSH) levels were within normal limits on his current dose of levothyroxine. A non-contrast CT head revealed no acute intracranial findings, excluding stroke. Initial EKG without any dynamic ischemic changes and decreased troponin levels after peaking at 0. 173 ng/mL reasoned out an acute ischemic event. Additional laboratory tests revealed acute-on-chronic renal failure, with urea nitrogen levels of 75 mg/dL, creatinine levels of 2.80 mg/dL (baseline: 1.5-1.8), and potassium levels of 6.7 mEq/L. Bilateral renal ultrasound revealed normal echogenicity without hydronephrosis or renal calculi. AKI was thought to be caused by a combination of dehydration and worsened by home medications (quinapril and furosemide). Hyperkalemia is common when a potassium-sparing diuretic like spironolactone and angiotensin-converting enzyme inhibitors (ACEIs) like quinapril are combined. Although these findings pointed to AKI and hyperkalemia as potential causes of the patient's weakness and bradycardia, significant potassium elevation (typically >7) is required for pure hyperkalemia to cause bradycardia of this magnitude, and our patient's EKG did not show peaking of T-waves or QT interval shortening (Figure 1). Furthermore, bradycardia and carvedilol use raised the possibility of beta-blocker toxicity; however, the patient's family reported medication compliance and denied overuse of his beta-blocker medication. As a result, neither hyperkalemia nor beta-blocker toxicity was likely to be the only cause of this patient's symptoms, making BRASH syndrome the most likely cause in our case.

Mild cases of BRASH syndrome frequently respond to simple medical treatments like intravenous calcium and fluid resuscitation. In moderate cases, the treatment of BRASH syndrome focuses on aggressive hyperkalemia therapy and fluid resuscitation. Hyperkalemia can be managed by membrane stabilization, shifting potassium intracellularly, and kaliuresis or dialysis. Fluid resuscitation depends on the volume status as some patients may be profoundly volume depleted, whereas others may present with volume overload due to anuric renal failure. As suggested by Dr. Farkas, isotonic bicarbonate is a good initial choice for fluid resuscitation, as most patients with BRASH syndrome will have a combination of hyperkalemia and metabolic acidosis [1]. Traditional bradycardia management using the Advanced Cardiovascular Life Support (ACLS) algorithm, including atropine and cardiac pacing, may not succeed in patients with BRASH syndrome in more severe cases of shock [9]. Instead, IV calcium, epinephrine, or isoproterenol should be included in the bradycardia ACLS algorithm in BRASH syndrome [10]. Correction of volume status, management of hyperkalemia, and treatment of the underlying cause are critical in all grades of BRASH syndrome.

Learning points

BRASH syndrome stands for Bradycardia, Renal Failure, AV-nodal blockade, Shock, and Hyperkalemia. The underlying pathophysiologic mechanism is unknown, but a synergistic effect of hyperkalemia and AV-nodal blockers has been reported to cause bradycardia, which causes renal hypoperfusion leading to worsening hyperkalemia and cycling back to bradycardia. The key thing about BRASH syndrome is recognizing all the pieces. We have all seen these patients in the past before we necessarily called them BRASH. Most patients can be easily managed with aggressive hemodynamic support, hyperkalemia management, and overseeing renal function without requiring specialized treatments. On the other hand, sick patients with shock require a central line and an arterial line. IV calcium and epinephrine or isoproterenol should be part of the ACLS algorithm for Bradycardia in BRASH syndrome. 

## Conclusions

In our patient, who had a moderate BRASH syndrome, normal saline was used for fluid resuscitation, hyperkalemia was aggressively treated with Lokelma after initial membrane stabilization with IV calcium gluconate, and temporizing measures with Insulin+D50 and Albuterol inhaler were used. Finally, the underlying cause was addressed by discontinuing home medications that contributed to AKI and hyperkalemia, such as Non-steroidal anti-inflammatory drugs (NSAIDs), quinapril, and spironolactone. He was advised to stay hydrated.
